# Associations among blood biomarkers, clinical subtypes, and prognosis in Parkinson’s disease

**DOI:** 10.1016/j.prdoa.2025.100313

**Published:** 2025-03-11

**Authors:** Hideki Oizumi, Takafumi Hasegawa, Ichiro Kawahata, Tomoki Sekimori, Tomoko Totsune, Yoko Sugimura, Toru Baba, Kohji Fukunaga, Atsushi Takeda

**Affiliations:** a Department of Neurology, National Hospital Organization Sendai Nishitaga Hospital, 2-11-11, Kagitorihoncho, Taihaku-ku, Sendai, Miyagi 982-8555, Japan; b Department of Neurology, Tohoku University Graduate School of Medicine, 2-1, Seiryo-machi, Aoba-ku, Sendai, Miyagi 980-8575, Japan; c Department of CNS Drug Innovation, Tohoku University Graduate School of Pharmaceutical Sciences, 6-3, Aramaki, Aoba-ku, Sendai, Miyagi 980-8578, Japan; d Department of Molecular Genetics, Institute of Biomedical Sciences, Fukushima Medical University Graduate School of Medicine, Hikarigaoka, Fukushima City, Fukushima 960-1295, Japan; e Department of Cognitive and Motor Aging, Tohoku University Graduate School of Medicine, 2-1, Seiryo-machi, Aoba-ku, Sendai, Miyagi 980-8575, Japan

**Keywords:** Parkinson’s disease, Neurofilament light chain, Total tau, Ubiquitin carboxyl-terminal hydrolase L1, Fatty acid-binding protein 3

## Abstract

**Background:**

Early identification of the poor prognosis subtype by surrogate markers would be advantageous for selecting treatments for Parkinson’s disease (PD). The aim of the present study was to test whether plasma neurofilament light chain (NF-L), total tau (t-tau), ubiquitin carboxyl-terminal hydrolase L1 (UCH-L1), fatty acid-binding protein 3 (FABP3), and phosphorylated tau (p-tau) can be used as prognostic biomarkers in PD.

**Methods:**

In the present study, both retrospective and prospective studies were performed. Plasma samples at baseline from 81 PD patients were included in the prospective study. Plasma samples at baseline from 60 patients who underwent cognitive assessment were subjected to the hierarchical cluster analysis for a retrospective study.

**Results:**

On the basis of the results of the cluster analysis, patients were classified into three groups: groups (G)1, G2 and G3. Individuals in the G1 cluster, who had an older age at onset and were prone to early progression with dementia, had significantly greater plasma NF-L levels than those in the G3 cluster, who did not present with dementia at an early stage. A Cox proportional hazards regression model adjusted for age and sex revealed that high NF-L and UCH-L1 levels at baseline predicted the four future milestones (i.e., nursing care, dysphagia, wheelchair use, and repeated falls), and high plasma t-tau at baseline predicted future dysphagia.

**Conclusions:**

Although further studies with a larger number of patients will be required, plasma NF-L may be a useful biomarker for identifying the rapidly progressive subtype of PD, and plasma NF-L and UCH-L1 may serve as biomarkers of overall PD prognosis, whereas plasma t-tau could be a biomarker for future dysphagia in PD.

## 1 Introduction

Parkinson's disease (PD) is a progressive neurodegenerative disease that affects more than 1 % of the population over 65 years of age [1]. The histopathological hallmarks of PD are the preferential loss of dopaminergic neurons in the *substantia nigra* and the appearance of intracytoplasmic proteinaceous aggregates called Lewy bodies (LBs), which are composed mainly of hyperphosphorylated, misfolded alpha synuclein (αSyn) [2]. Aberrant αSyn aggregation in neurons is thought to be associated with selective neurodegeneration in PD and other synucleinopathies [3].

The clinical and pathological heterogeneity of PD is well known, but whether PD has different clinical entities or a single process involving a spectrum of disorders has been a matter of debate [4]. From the viewpoint of disease-modifying therapy (DMT), early identification of the poor prognosis subtype is advantageous because appropriate therapeutic interventions can be initiated earlier in certain patients, which ultimately leads to improve the management of daily life in PD. Therefore, there is an urgent need to develop surrogate markers that can accurately predict the prognosis of this disease. Using cluster analysis based on clinical symptoms and histopathological characteristics suggested, a group of researchers suggested that there are three subgroups of PD: the diffuse malignant type, intermediate type, and mild–motor predominant type [5]. The diffuse malignant type has a poor prognosis, with rapid progression irrespective of the stage of the underlying LB and/or Alzheimer's disease (AD) pathology, and patients with this PD type have a higher age at death than the other groups do [5]. Similarly, we recently performed data-driven subtyping of PD on the basis of ^123^I-metaiodobenzylguanidine (MIBG) cardiac scintigraphy, dopamine transporter (DAT) imaging data and disease duration data and reported that the rapidly progressive type presented diffuse cerebral atrophy and marked loss of cardiac sympathetic nerve terminals from the early stage of the disease [6].

Neurofilament light chain (NF-L) is a component of large myelinated axons that is known to be elevated not only in cerebrospinal fluid (CSF) but also in blood following neuroaxonal injury. NF-L has been recognized as a biomarker for a variety of neurogenerative diseases and traumatic brain injury [7]. Like NF-L, fatty acid-binding protein 3 (FABP3), a member of the lipid chaperone family expressed in the brain as well as in the heart, is elevated in the peripheral blood of PD patients [8,9]. In a previous study, we found that FABP3 is involved in the aggregation and propagation of αSyn, which is a cause of PD [10,11]. Furthermore, in subsequent studies using patient blood, we revealed that plasma NF-L and FABP3, along with other plasma neurodegenerative-related proteins, such as UCH-L1 and t-tau, would be useful for the differential diagnosis of LB diseases, including PD and dementia with LB (DLB) [12]. In previous study, we have confirmed that the levels of NF-L, UCH-L1, and FABP3 in plasma were higher in PD patients than those of controls [12]. In support of this notion, elevated levels of NF-L and FABP3 in CSF have been shown to be predictive of future dementia in PD patients [13]. However, the predictive value of plasma NF-L and FABP3, along with other plasma neurodegenerative-related proteins, such as UCH-L1 and t-tau remains uncertain. To address this issue, we performed an ultrasensitive biomarker assay using a single-molecule array (SIMOA) assay, hierarchical cluster analysis on the basis of clinical symptoms for a retrospective study, and a Cox proportional hazards regression model and Kaplan–Meier curve analysis for a prospective study to investigate whether plasma neurodegenerative-related proteins could be utilized as prognostic markers in PD.

## 2 Methods

***Clinical information of the participants in the present study***

All participants were recruited at the National Hospital Organization Sendai Nishitaga Hospital and examined by board-certified neurologists. Patients were recruited from October 2017 to March 2020, and 81 patients who were diagnosed with clinically established PD according to the Movement Disorder Society clinical diagnostic criteria were enrolled (Table S1) [14]. The exclusion criteria at baseline were defined as having 4 milestones [i.e., nursing care (NC), dysphagia, wheelchair use (WC), and repeated falls (falls)], stage 5 at the Hoehn and Yahr (HY) scale, and using antipsychotic drugs. The patients included 33 males and 48 females. The age ranged from 49–92 years, with a mean of 73.5 years, and the duration of disease ranged from 0–29 years, with a mean of 7.8 years. The duration of disease was defined as the period since the onset of motor symptoms. All patients had idiopathic PD, were treated with L-DOPA alone or with other antiparkinsonian drugs and had well-controlled motor symptoms. The motor function assessments by the HY staging were performed during on-time periods. Likewise, the cognitive assessments using the Mini-Mental State Examination (MMSE) and the Japanese version of the Montreal Cognitive Assessment (MoCA-J) were conducted during on-time periods. Plasma samples and clinical manifestations at baseline from 81 PD patients were included in the prospective study (Fig. S1). Plasma samples and clinical manifestations at baseline from 60 patients who underwent cognitive tests were included in the hierarchical cluster analysis for a retrospective study (Fig. S1). All patients received repeated motor and cognitive assessments during the follow-up periods. The observation period ranged from 3-6 years, with a mean of 5.02 years. Since the main purpose of the present study was to discriminate the two PD subtypes, I.e., rapidly progressive and slowly progressive subtypes, using plasma biomarkers, we did not include control cases. The present study was approved by the ethics committee of Sendai Nishitaga Hospital (approval no. R5-8). All participants provided informed consent in accordance with the guidelines of the Declaration of Helsinki. Throughout the study, ethical considerations were taken into account, including patient consent and data privacy.

***Sample collection***

Plasma samples at baseline were collected on the same day as the cognitive and motor function assessments. Sample collection was performed from October 2017 to March 2020. Briefly, fasting blood was collected in a test tube containing Na-EDTA and centrifuged at room temperature for 10 min to isolate the plasma fraction. The plasma was then divided into 500 μl aliquots and collected in screw-cap microtubes (Sarstedt AG, Nümbrecht, Germany) between 10 and 12 am. All samples were stored at −80 °C before the SIMOA assay.

***SIMOA assay***

The SIMOA assay was performed on plasma samples according to a previously reported method [12]. In brief, the samples stored at −80 °C were thawed and centrifuged at 10,000*g* for 5 min. To confirm the performance characteristics of the SIMOA assay, a calibration curve was created using standard material for each biomarker (data not shown). Prior to the experiments, the average of two measurements using each calibrator was used to create the calibration curve. After the calibration curve was created, each sample was measured once. SIMOA-based quantification of neurodegeneration-related proteins was conducted with the Simoa HD-X system^TM^ (Quanterix, Billerica, MA, USA). Prior to the assay, the samples were diluted with the sample diluent provided in each assay kit or in the Homebrew Assay Development Kit (Quanterix). The diluted samples were then applied to target-specific antibody-immobilized plates and assayed individually. The subsequent measurements were performed according to the manufacturer’s instructions (Quanterix). The Simoa Neurology 4-Plex A Kit (#102153; Quanterix) was used to measure the concentrations of t-tau, NF-L, and UCH-L1, and the Simoa p-Tau181 Advantage Kit (#103377; Quanterix) was used to quantify phosphorylated tau (p-tau) at residue 181. To measure FABP3 levels, a custom assay system was developed using the Homebrew Assay [12], and the assay was conducted according to the protocol provided with the Simoa Homebrew Assay Development Kit (#101354, Quanterix). The reagents for the assay were prepared with Pierce EDC (#A35391; Thermo Fischer Scientific, Waltham, MA, USA) and an EZ-Link NHS-PEG4-Biotin kit (#A39259; Thermo Fischer Scientific). Capture antibodies were coated onto magnetic beads at a concentration of 0.3 mg/mL, and the detector antibodies were biotinylated at a 1:40 ratio. In the pilot study, these customized reagents ensured specific detection of FABP3 in the samples. Note that the examiner was blinded to the sample group assignments.

### 2.1 Statistical analysis

In present study, we performed both retrospective and prospective studies, with hierarchical cluster analysis being used for the former, and Kaplan–Meier curves and Cox proportional hazards regression models being used for the latter.

The data of sixty PD patients who underwent cognitive function tests were analyzed via cluster analysis. Specifically, hierarchical cluster analysis was performed with the Ward linkage method to classify patients on the basis of MMSE score, age at onset, and disease duration (years). Comparisons of clinical manifestations, demographic features, and plasma neurodegenerative-related proteins among the classified clusters were performed via one-way ANOVA with Bonferroni *post hoc* correction, the nonparametric Kruskal–Wallis test with Bonferroni *post hoc* correction, or the chi–square test, depending on the parameters.

All 81 PD patients with baseline plasma samples available were included in the prospecive study. Using Kaplan–Meier curves and Cox proportional hazards regression models, we established four milestones [i.e., NC, dysphagia, WC, and falls] as indicators of advanced-stage disease. At follow-up, two patients who died from causes unrelated to PD prior to NC; 10 patients who had falls at the time of participation; and one patient with missing plasma FABP3, t-tau, and UCH-L1 data were excluded from each prospective study. A Cox proportional hazards regression model was used to compare two groups; the group pairs included the high and low groups of NF-L, t-tau, UCH-L1, FABP3, and p-tau (phosphorylated at residue 181) levels. For this analysis, the cutoff values for plasma NF-L, t-tau, UCH-L1, FABP3, and p-tau at baseline were set as follows: ROC (receiver operating characteristic) curve analysis with and without the four milestones was performed, and the candidate cutoff level was determined based on the highest Youden index and the proportional assumption. Kaplan–Meier curves were used to compare the cumulative risk between the following groups: the high and low groups of NF-L, UCH-L1, and t-tau levels. In the Kaplan–Meier curve analysis of the cumulative risk of disease milestones, the cutoff values for plasma NF-L, UCH-L1, and t-tau at baseline were set as follows: ROC curve analysis with and without the four milestones was performed, and the cutoff level with the highest Youden index was determined. According to the Kaplan–Meier curve analysis, differences in the cumulative risk of the four future milestones between the high and low plasma protein groups at baseline were analyzed via the log-rank test. One-way ANOVA with Bonferroni *post hoc* correction, the nonparametric Kruskal–Wallis test with Bonferroni *post hoc* correction, and time-dependent Cox regression analysis were performed with IBM SPSS Statistics 28 software (IBM SPSS, Armonk, NY). Cluster analysis, chi-square tests, Cox proportional hazards regression models and Kaplan–Meier curves were generated via JMP15 software (SAS Institute, Tokyo, Japan). The data are expressed as the means ± standard deviations. A P value less than 0.05 was considered to indicate statistical significance.

## 3 Results

***Cluster analysis***

**Fig. 1:**
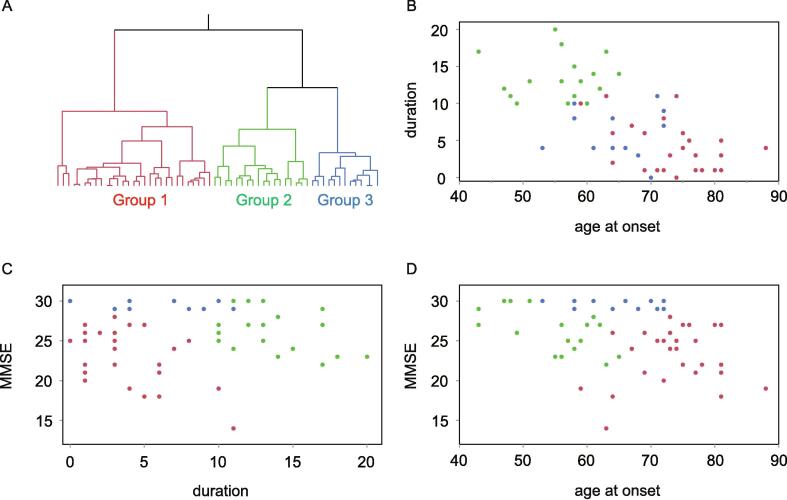
Dendrogram of the unbiased hierarchical cluster analysis solution and two-dimensional (2D) plots of the clusters. (A) A dendrogram of hierarchical cluster analysis. (B, C, and D) 2D plots of MMSE score, age at onset, and disease duration on the two axes, respectively. The G1, G2, and G3 clusters are shown as red, green, and blue dots, respectively.

A dendrogram was obtained via unbiased hierarchical cluster analysis using 60 patients who underwent cognitive tests (Fig. 1A). Fig. 1B-D represents two-dimensional plots of data with each parameter (i.e., MMSE score, age at onset, and disease duration) on two axes, in which the G1, G2, and G3 clusters are indicated by red, green, and blue dots, respectively. Intriguingly, both G1 and G3 had relatively early disease onset and similar clinical characteristics in terms of disease duration and HY stage (Fig. 1B and 1C, Table 1). However, G1 had lower cognitive test scores (MMSE, MoCA-J), which can be interpreted as indicators of advanced disease stage, and a greater age at onset than G3 did (Fig. 1C and 1D, Table 1). Moreover, G2 had a longer disease duration and a greater levodopa equivalent daily dose (LEDD) than the other groups did (Fig. 1B and 1C, Table 1). Compared with G3, G2 presented slightly lower cognitive function and greater motor dysfunction (HY stage) (Fig. 1C and 1D, Table 1). Notably, the frequency of antidementia medication (ADM) use did not differ significantly between the groups, suggesting that the effect of ADMs on cognitive function was negligible in each group (Table 1). Subsequent nonparametric Kruskal–Wallis tests of plasma NF-L levels in each cluster revealed that plasma NF-L levels were significantly greater (P < 0.05) in G1 than in G3, whereas comparisons between G1 and G2 and between G2 and G3 did not reveal significant differences (Table 1). Nonparametric Kruskal–Wallis tests of plasma t-tau, UCH-L1, FABP3, and p-tau levels in each cluster revealed that comparisons between each group did not reveal significant differences (Table 1). We speculated that the variability of biomarker levels within each cluster could be due to the clinical characteristics, such as cognitive and motor function.

**Table 1:** Clinical manifestations, demographic features, and plasma biomarkers in PD patients analyzed via cluster analysis.

Characteristics	All PD(n = 60)	G1(n = 29)	G2(n = 18)	G3(n = 13)	P value	G1 vs. G2	G1 vs. G3	G2 vs. G3
Sex (n)					0.17			
Male	24	15	6	3				
Female	36	14	12	10				
Age (y)	74 ± 7.6	78.3 ± 5.6	69.3 ± 7.3	70.7 ± 6.9	**< 0.001**	**< 0.001**	**< 0.01**	1
Age at onset (y)	66.2 ± 10.4	73.8 ± 6.4	55 ± 6.7	64.5 ± 6.1	**< 0.001**	**< 0.001**	**< 0.001**	**< 0.001**
Duration (y)	7.3 ± 5.3	3.9 ± 3.2	13.7 ± 3	5.8 ± 3.2	**< 0.001**	**< 0.001**	0.19	**< 0.001**
HY	2.7 ± 0.8	2.45 ± 0.7	3.2 ± 0.8	2.38 ± 0.7	**< 0.01**	**< 0.01**	1	**< 0.05**
MMSE (pt)	25.5 ± 3.7	23.2 ± 3.2	26.1 ± 2.6	29.5 ± 0.5	**< 0.001**	**< 0.001**	**< 0.001**	**< 0.01**
MOCAJ (pt)	21.5 ± 4.4	18.7 ± 3	22.5 ± 3.9	26 ± 3	**< 0.001**	**< 0.01**	**< 0.001**	**< 0.05**
LEDD (mg)	874 ± 514	665 ± 391	1306 ± 500	743 ± 428	**< 0.001**	**< 0.001**	0.51	**< 0.01**
ADM, n (%)	10 (17)	5 (17)	4 (22)	1 (8)	0.56			
NF-L (pg/ml)	25.4 ± 14.4	28.6 ± 14.6	24.7 ± 13	19.3 ± 14.6	**< 0.05**	0.77	**< 0.05**	0.32
t-tau (pg/ml)	0.8 ± 0.5	0.8 ± 0.6	0.8 ± 0.4	0.6 ± 0.4	0.25			
UCH-L1 (pg/ml)	28.4 ± 12.8	31 ± 13.6	25.5 ± 10.6	26.8 ± 13.4	0.23			
FABP3 (ng/ml)	4.5 ± 3	4.7 ± 2.8	4.4 ± 3.2	4 ± 3.2	0.21			
p-tau (pg/ml)	1 ± 2.2	1.4 ± 2.5	0.5 ± 2.2	0.7 ± 1.4	0.12			

Abbreviations: ADM: anti-dementia medications; FABP3: fatty acid-binding protein 3; G: group; HY: Hoehn and Yahr stage; LEDD: levodopa equivalent daily dose; MMSE: mini mental state examination; MOCAJ: Japanese version of the Montreal cognitive assessment; NF-L: neurofilament light chain; p-tau: phosphorylated tau; PD: Parkinson’s disease; t-tau: total tau; UCH-L1: ubiquitin carboxy-terminal hydrolase L1; y: year.

Notes:

Comparisons of consecutive clinical demographic data among the subtypes were performed via one-way analysis of variance with a post hoc Bonferroni correction.

Comparisons of plasma parameters among the subtypes were performed via the nonparametric Kruskal–Wallis test with post hoc Bonferroni correction.

Comparisons of sex among the subtypes were performed via the chi-square test.

***Kaplan***–***Meier curves and Cox proportional hazards regression model***

All 81 patients whose baseline plasma samples were available were included in the prospective follow-up study. During the observation period (3–6 years), NC, dysphagia, WC, and falls occurred in 38 (48 %), 17 (21 %), 51 (63 %), and 60 (85 %) patients, respectively. The median times from the start of the study to the appearance of NC, dysphagia, WC, and falls were 4.25, 4.33, 3.67, and 2.95 years, respectively.

The univariate Cox proportional hazards regression model revealed that high plasma NF-L and UCH-L1 levels at baseline significantly contributed to an increased risk for the aforementioned four milestones, whereas high plasma t-tau and FABP3 levels at baseline significantly increased the risk for three milestones (NC, dysphagia, and WC) in this univariate model (Table 2). A high plasma p-tau level at baseline significantly increased the risk ratio for falls in this univariate model (Table 2). Moreover, in the multivariate Cox proportional hazards regression model adjusted for age and sex, a high plasma NF-L concentration at baseline was significantly associated with future NC (hazard ratio (HR), 3.7; 95 % CI, 1.6–8.4; P < 0.01), dysphagia (HR, 4.9; 95 % CI, 1.5–15.6; P < 0.01), WC (HR, 2.9; 95 % CI, 1.5–5.5; P < 0.01), and falls (HR, 2.3; 95 % CI, 1.2–4.5; P < 0.05) (Table 2). Notably, in this multivariate model, a high plasma t-tau concentration at baseline was significantly correlated with an increased risk for future dysphagia (HR, 6.4; 95 % CI 1.4–29; P < 0.05) (Table 2). Furthermore, the groups with high plasma UCH-L1 levels at baseline presented significantly greater risk ratios for NC (HR, 2.8; 95 % CI 1.3–6; P < 0.01), dysphagia (HR, 3; 95 % CI 1.1–8.3; P < 0.05), WC (HR, 2; 95 % CI 1.1–3.7; P < 0.05), and falls (HR, 2.3; 95 % CI 1.2–4.3; P < 0.05) (Table 2). Overall, even in this multivariate model adjusted for age and sex, high plasma NF-L and UCH-L1 levels were predictive of the aforementioned four milestones, and high t-tau levels may predict future dysphagia.

**Table 2:** Plasma biomarkers related to the clinical prognosis of PD according to the Cox proportional hazards regression model.

Variable	Univariate hazard ratio (95 % CI)	P value	Multivariate hazard ratio (95 % CI)	P value
Higher risk of NC				
NF-L > 23 pg/ml	5 (2.4–10.6)	**< 0.0001**	3.7 (1.6–8.4)	**< 0.01**
t-tau > 0.745 pg/ml	2.2 (1.2–4.3)	**< 0.05**	1.4 (0.7–2.8)	0.29
UCH-L1 > 22.48 pg/ml	3.1 (1.5–6.3)	**< 0.01**	2.8 (1.3–6)	**< 0.01**
FABP3 > 4.16 ng/ml	2.7 (1.4–5.2)	**< 0.01**	1.8 (0.8–3.9)	0.14
p-tau > 0.2 pg/ml	1.9 (1–3.7)	0.068		
Higher risk of dysphagia				
NF-L > 27.8 pg/ml	6.8 (2.4–19.5)	**< 0.001**	4.9 (1.5–15.6)	**< 0.01**
t-tau > 0.745 pg/ml	8.4 (1.9–37.1)	**< 0.01**	6.4 (1.4–29)	**< 0.05**
UCH-L1 > 34 pg/ml	4.1 (1.5–11.1)	**< 0.01**	3 (1.1–8.3)	**< 0.05**
FABP3 > 4.31 ng/ml	2.9 (1.1–7.7)	**< 0.05**	1.5 (0.5–4.8)	0.45
p-tau > 0.05 pg/ml	2.4 (0.9–6.4)	0.067		
Higher risk of WC				
NF-L > 24.2 pg/ml	3.2 (1.8–5.7)	**< 0.0001**	2.9 (1.5–5.5)	**< 0.01**
t-tau > 0.745 pg/ml	2.5 (1.4–4.4)	**< 0.01**	1.8 (1–3.3)	0.053
UCH-L1 > 23 pg/ml	2 (1.1–3.7)	**< 0.05**	2 (1.1–3.7)	**< 0.05**
FABP3 > 3.8 ng/ml	2 (1.2–3.6)	**< 0.05**	1.8 (1–3.3)	0.06
p-tau > 0.05 pg/ml	1.7 (0.9–3)	0.075		
Higher risk of falls				
NF-L > 24.3 pg/ml	2.1 (1.2–3.7)	**< 0.01**	2.3 (1.2–4.5)	**< 0.05**
t-tau > 0.745 pg/ml	1.7 (0.9–2.9)	0.077		
UCH-L1 > 22.4 pg/ml	2.1 (1.1–3.7)	**< 0.05**	2.3 (1.2–4.3)	**< 0.05**
FABP3 > 4.58 ng/ml	1.7 (1–3.1)	0.051		
p-tau > 0.05 pg/ml	2.1 (1.2–3.8)	**< 0.05**	1.9 (1.1–3.5)	**< 0.05**

Abbreviations: FABP3: fatty acid-binding protein 3; Falls: repeated falls; NC: nursing care; NF-L: neurofilament light chain; PD: Parkinson’s disease; p-tau: phosphorylated tau; t-tau: total tau; UCH-L1: ubiquitin carboxy-terminal hydrolase L1; WC: wheelchair use.

Notes:

Baseline plasma biomarker levels were used as dichotomized predictor variables. In the multivariate analysis, adjustments were made for age and sex.

**Fig. 2:**
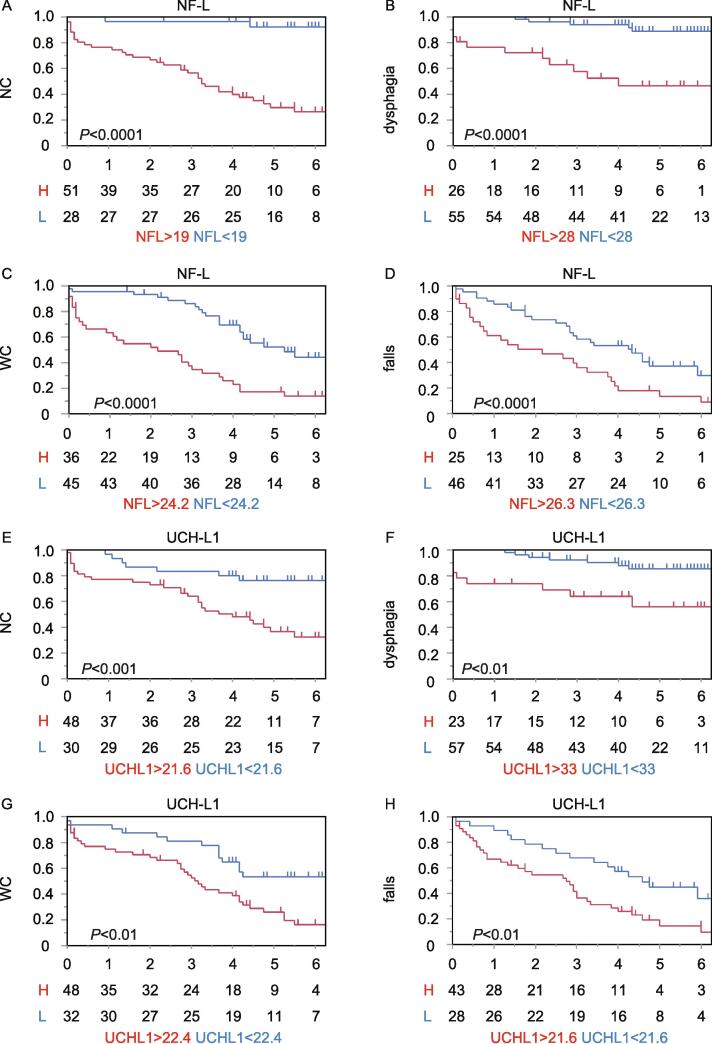
Relationships between plasma biomarker levels and the future risk of the four milestones. (A-D) Kaplan–Meier curves of the cumulative risk of NC, dysphagia, WC and falls in the high- and low-plasma NF-L groups. (E-H) Kaplan–Meier curves of the cumulative risk of NC, dysphagia, WC and falls in the high- and low-plasma UCH-L1 groups. P values were analyzed via the log-rank test. Abbreviations: H, high; L, low.

Kaplan–Meier curve analysis of the cumulative risk of NC (P < 0.0001), dysphagia (P < 0.0001), WC (P < 0.0001), and falls (P < 0.0001) revealed that the groups with high plasma NF-L levels at baseline had a significantly greater incidence (Fig. 2A–D). Similarly, regarding the cumulative risk of NC (P < 0.001), dysphagia (P < 0.01), WC (P < 0.01), and falls (P < 0.01), patients with high plasma UCH-L1 levels at baseline had a significantly greater incidence (Fig. 2E-H). Kaplan–Meier curve analysis of the cumulative risk of dysphagia (P < 0.001) revealed that the groups with high plasma t-tau levels at baseline had a significantly greater incidence (Fig. S2).

## 4 Discussion

Braak’s hypothesis states that the pathological process of PD initially occurs in the lower brainstem and subsequently spreads into the limbic and cortical areas in a caudo-rostral fashion. However, whether this concept is applicable to all subtypes of PD has been a topic of debate [15,16]. To date, numerous subtypes based on motor and nonmotor symptoms, including the postural instability and gait disorders (PIGD) subtype, have been proposed to identify individuals with a rapid course of PD with a poor prognosis [5,17]. A recent study subtyping PD on the basis of the severity of clinical symptoms and neuropathological findings demonstrated that Braak’s ascending scheme can explain the early-onset, slowly progressive subtype (known as the “mild-motor predominant type”), but it does not apply to the late-onset, rapidly progressive subtype (the so-called “diffuse malignant type”) [5]. Similarly, in our cluster analysis based on nuclear imaging data from MIBG cardiac scintigraphy and DAT imaging together with clinical features, a caudo-rostral gradient in the severity of imaging findings was observed in the early-onset, slowly progressive type (i.e., mild–motor predominant type) [5,6]. In contrast, in the rapidly progressive type, marked cortical atrophy was observed concomitant with the loss of cardiac sympathetic nerve terminals and decreased striatal DAT binding, corresponding to the diffuse malignant phenotype described above [5,6]. Although accumulating evidence from these clinical, imaging, and pathological aspects is enabling more accurate stratification of PD subtypes, no studies have examined the correlations between disease subtypes and easily accessible blood biomarkers.

Consistent with our previous observations [6], the subtype analysis of PD based on clinical findings in this study also identified three clusters: G1, G2 and G3. Compared with G3, G2 presented milder cognitive and motor dysfunction despite a longer duration of age. However, both clusters had similar ages at onset that were younger than those in G1. Thus, G1 has different characteristics from G2 and G3, suggesting that G1 represents a single subtype. More specifically, G1, G2, and G3 could be defined as clusters corresponding to the rapidly progressive type at the early stage (RPT-E), slowly progressive type at the late stage (SPT-L), and slowly progressive type at the early stage (SPT-E), respectively, and the prognosis of G1 seems to be poorer than that of G2 and G3.

The identification of biomarkers indicating poor prognosis in PD subtype groups will contribute to personalized therapy when future therapeutic interventions that halt or slow disease progression become available. Thus, we compared plasma neurodegeneration-related proteins in each subtype obtained from cluster analysis. The finding of elevated plasma NF-L levels in G1 patients compared with those in G3 patients corresponds well with the previous notion that elevated plasma NF-L levels are indicative of a poor prognosis [7]. Importantly, in our cohort, high plasma NF-L levels at baseline could predict four milestones (NC, dysphagia, WC, and falls). Our finding is consistent with a previous study demonstrating that high level of NF-L at baseline could predict future NC and motor dysfunction in PD [18]. This prospective study also showed that high NF-L level could predict future dementia and death [18]. Interestingly, another report suggested that high level of NF-L could also predict future depression [19]. Although the exact pathological process of PD remains elusive, it has been postulated that αSyn aggregates initially occur in nerve endings, followed by axonal damage [[20], [21], [22]]. Then, Lewy bodies (LBs) are formed in the soma [[20], [21], [22]]. This finding likely indicates that elevated blood NF-L levels may reflect early neuroaxonal damage and thus may be useful as a surrogate marker for a poor prognosis of PD.

Importantly, we also found that at baseline, high UCH-L1 levels in plasma samples could predict the aforementioned four milestones in PD. Note that there have been no previous studies showing a relationship between blood or CSF UCH-L1 levels and prognosis of PD. UCH-L1 is the causative molecule of the rare familial form of PD (PARK5) and is highly expressed in neural tissues, accounting for 1–2 % of total brain protein [23]. Like parkin, the most common cause of autosomal recessive juvenile parkinsonism, UCH-L1 plays a key role in the ubiquitin–proteasome system, acting as a deubiquitinase enzyme [24]. Since UCH-L1 and αSyn are known to be colocalized in LBs, this protein might also be involved in the initiation and progression of sporadic PD [25]. Although somewhat provocative, the blood UCH-L1 level might be related to the brain burden of LB pathology.

Plasma biomarker has the advantages of being minimally invasive and relatively low cost. In combination with other diagnostic modalities including neuroradiological imaging, it is expected to enable earlier therapeutic interventions, eventually improving a quality of life of PD.

## 5 Limitations of the present study

The present study has several limitations. First, patients were not diagnosed pathologically. In the next scrutiny, we would like to make pathological diagnosis of the participants to enhance the credibility of the study. Second, several potential biases are suggested: (1) The present study was conducted at a single center, and the sample size was not enough. (2) Patients who were more severely affected tended to be included. (3) Plasma NF-L levels were significantly greater in G1 than in G3, but the statistical power between G1 and G3 for plasma NF-L was low (0.46). Collecting de novo cases over time at multiple centers could contribute to the generalizability of the study. Third, the present study lacked clinical data, such as Unified Parkinson's Disease Rating Scale scores. However, the main focus of the present study was a biomarker survey for the prognosis of PD, and we considered that minimal clinical information should be available. Fourth, the present study lacked performing biomarker measurements over time. In the future, we would like to collect plasma samples over time to further examine the correlation between the amount of changes in biomarker and clinical outcome.

## 6 Conclusions

Plasma NF-L may be a useful biomarker for identifying the rapidly progressive subtype of PD, whereas plasma NF-L, UCH-L1, and t-tau may also contribute to the prognostic prediction of PD at different stages. It should be emphasized that the present study had extensive follow-up period, averaging of 5.02 years. Although further validation in more patients is needed, these neurodegenerative-related proteins in plasma could be useful for PD subtype stratification and prognostic prediction. If plasma biomarkers can predict adverse events such as fracture due to falls and aspiration pneumonia in advance, they may contribute to more effective and earlier care planning. Furthermore, it has been shown that αSyn antibody therapy, one of the DMTs for PD, would be more effective in the rapidly progressing, diffuse malignant subtype of PD [26]. By combining plasma biomarkers with other diagnostic modalities, it may be possible to choose patients who are more likely to respond to DMT.

Funding

This work was supported by grants-in-aid for Scientific Research from the Project of Translational and Clinical Research Core Centers from the Japan Agency for Medical Research and Development (AMED) grant numbers JP17dm0107071, JP18dm0107071, JP19dm0107071, and JP20dm0107071 (K.F. and A.T.); the Research Committee of CNS Degenerative Diseases, Research on Policy Planning and Evaluation for Rare and Intractable Diseases, Health, Labor and Welfare Sciences Research Grants, the Ministry of Health, Labor and Welfare, Japan (A.T.); the Japanese Ministry of Education, Culture, Sports, Science and Technology, Scientific Research (C) grant number 23K06823 (T.H.); the Japan Society for the Promotion of Science (JSPS) WISE Program: Advanced Graduate Program for Future Medicine and Health Care (24KJ0359) (T.S.); and the Japan Agency for Medical Research and Development (AMED) grant number 22ym0126095h0001 and 23ym0126095h0002), JSPS Grants-in-Aid for Scientific Research (KAKENHI, grant number 22K06644), and the Takeda Science Foundation (I.K.).

## CRediT authorship contribution statement

**Hideki Oizumi:** Writing – original draft, Validation, Resources, Formal analysis, Data curation, Conceptualization. **Takafumi Hasegawa:** Writing – review & editing, Writing – original draft, Visualization, Supervision, Resources, Funding acquisition, Data curation, Conceptualization. **Ichiro Kawahata:** Funding acquisition, Formal analysis, Conceptualization. **Tomoki Sekimori:** Funding acquisition, Formal analysis. **Tomoko Totsune:** Resources. **Yoko Sugimura:** Resources. **Toru Baba:** Resources. **Kohji Fukunaga:** Supervision, Funding acquisition, Conceptualization. **Atsushi Takeda:** Writing – review & editing, Visualization, Supervision, Resources, Funding acquisition, Data curation, Conceptualization.

## Declaration of competing interest

The authors declare that they have no known competing financial interests or personal relationships that could have appeared to influence the work reported in this paper.
