# Supplementary material for: Associations among blood biomarkers, clinical subtypes, and prognosis in Parkinson’s disease
Source: Clin Park Relat Disord. 2025 Mar 11;12:100313. doi: 10.1016/j.prdoa.2025.100313 (PMC11952764; doi:10.1016/j.prdoa.2025.100313)
Supplement: Supplementary Data 1 [file mmc1.docx]

**Table S1.** Clinical manifestations, demographic features, and plasma biomarkers of all PD patients

| Characteristics | Patients (n = 81) |
| --- | --- |
| Sex (n) |  |
| Male | 33 |
| Female | 48 |
| Age (y) | 73.5 ± 8.5 |
| Age at onset (y) | 65.2 ± 11.3 |
| Duration (y) | 7.8 ± 6.3 |
| HY (pt) | 2.7 ± 0.8 |
| MMSE (pt) | 25.5 ± 3.7 |
| MOCAJ (pt) | 21.5 ± 4.4 |
| NF-L (pg/ml) | 27.6 ± 16.6 |
| t-tau (pg/ml) | 0.8 ± 0.5 |
| UCH-L1 (pg/ml) | 27.9 ± 13.7 |
| FABP3 (ng/ml) | 4.5 ± 3.1 |
| p-tau (pg/ml) | 0.9 ± 2.2 |

Abbreviations: FABP3: fatty acid-binding protein 3; HY: Hoehn and Yahr stage; MMSE: mini-mental state examination; MOCAJ: Japanese version of the Montreal cognitive assessment; NF-L: neurofilament light chain; PD: Parkinson’s disease; p: point; p-tau: phosphorylated tau; t-tau: total tau; UCH-L1: ubiquitin carboxy-terminal hydrolase L1; y: -year.
